# The Effect of Group-Based Occupational Therapy on Performance and Satisfaction of Stroke Survivors: Pilot Trail, Neuro-Occupational View

**DOI:** 10.15412/J.BCN.03080109

**Published:** 2017-01

**Authors:** Maryam Mehdizadeh, Afsoon Hassani Mehraban, Roohollah Zahediyannasab

**Affiliations:** 1. Department of neuroscience, School of Advanced Technologies in Medicine, Iran University of Medical Sciences, Tehran, Iran.; 1. Department of Occupational Therapy, Rehabilitation Research Center, School of Rehabilitation Sciences, Iran University of Medical Sciences, Tehran, Iran.; 3. Department of Occupational Therapy, School of Rehabilitation Sciences, Shiraz University of Medical Sciences, Shiraz, Iran.

**Keywords:** Stroke, Performance and satisfaction, Neuro-occupational

## Abstract

**Introduction:**

Stroke imposes limitations on performing activities of daily living (ADL) and their level. Different therapeutic approaches are used for improving the level of performance after a stroke. This study was performed with the aim of evaluating the effect of group-based occupational therapy on improving the performance of ADL and satisfaction of its performance in patients with chronic strokes.

**Methods:**

Fourteen chronic stroke patients with the mean age of 52 years participated in the study. The participants were assigned into two groups (control and treatment). The level of performance of ADL, level of stroke disability, and participation were respectively evaluated by Barthel index (BI), modified Rankin scale (MRS), and Canadian Occupational Performance Measure (COPM). Six sessions of group therapy tasks were scheduled with an emphasis on three main activities, including mobility exercises, craft, and cooking.

**Results:**

The COPM changes in the ‘performance’ and ‘satisfaction’ scores in the treatment group and the ‘performance’ scores in the control group were significant. The MRS scale in the two groups revealed no change in the level of stroke disability. However, the changes in the ADL performance in BI were significant.

**Conclusion:**

The current study indicated that doing daily, craft, and mobility activities in the groups can affect the ‘performance’ and ‘satisfaction’ levels in stroke patients.

## 1. Introduction:

Stroke is a complex dysfunction that originates from a lesion in the brain and is characterized by sudden onset of neurological defects. These defects are caused by vascular damage in the brain, resulting in partial or complete paralysis of half or the whole body (Gillen et al., 2006; Go et al., 2014). About 70–80 percent of people with stroke are experiencing restrictions on the movements of their upper and lower limbs. These limitations may actually cause difficulties in performing activities of daily living (ADL) and reducing the level of activity and mobility. Six months after stroke, 25–53 percent of them are dependent in at least one of the daily living tasks (Pang et al., 2006; Legg et al., 2007). Different therapeutic approaches for improving the level of limbs function and mobility after stroke are used, such as training and functional exercises, muscle strengthening, task-oriented exercises, Proprioceptive neuromuscular facilitation, sensory-motor and community-based services (Egan et al., 2007; KO et al., 2015; Urton et al., 2007).

Most of the time in rehabilitation centers these approaches are used individually and one by one. One of the approaches that has been recently came up to restore the level of performance and mobility in people with chronic stroke, is circuit class therapy (CCT) (Hillier et al., 2011; English et al., 2011; English et al., 2007). In this therapeutic method, more than two clients (in accordance with group therapy) are attending in the therapy session at the same time. Given tasks are according to each person’s level of performance. The main emphasis of this process is on doing the functional activities repeatedly to involve individuals in total therapy sessions (Hillier et al., 2011). Due to the dynamic and participation of all clients in group-based therapy, this method can increase the patient’s motivation, activity and social mobilization, so it can improve the quality of life of these people eventually (English et al., 2011).

According to the holistic and client-centered approach in occupational therapy; active participation during therapy sessions and satisfaction of how to do the tasks, the group is also have raised to provide health services (Jongbloed et al., 1991; Wressle et al., 2002; Morgan et al., 2002). The theoretical model of neuro-occupation is the concept that inspired by the system theory and was created from the combination of two concepts; neuroscience and occupation. It shows the non-linear nature in order to justify the relationship between the nervous system and the participation of people in occupation. Neuro-occupation is interdependent relationship between brain, nervous system and intention to restore or seek meaningful occupation (Derakhshanrad et al., 2015; Gutman and Biel, 2001).

Neuro-occupation is the concept to justify the voluntary participation of individual in activities which leads to the formation of meaning for participant. According to this model, the type of occupation chosen by the individual and the environment in which it operates can affect the brain function (Lazzarini et al., 2004). Improving the level of performance is one of the main goals of occupational therapy in patients with chronic stroke to enhance the independence, mobility and increased participation in the community and finally, improvement in the quality of life in these clients (Legg et al., 2007; Egan et al., 2007; English et al., 2011). This study investigates the effect of group therapy on the level of performance of activities of daily living and patient’s satisfaction chronic stroke with Neuro-occupational view.

## 2. Methods

### 2.1. Participants

In this experimental study fourteen stroke patients (9 men and 5 women with a mean age of 52±10.6 years and mean time of 18 months after the stroke) who received occupational therapy services on a daily basis participated. People were assigned into two groups (control/treatment) according to referral to two outpatient OT clinic. Criteria for inclusion include; occurrence of cerebrovascular accident which led to unilateral hemiplegia, the ability to walk independently at least with cane and no history of other neurological diseases. People had chronic cerebrovascular accident (more than 6 months). Before the stroke, these people had the ability to perform activities of daily living independently. In terms of cognitive level, participants had MMSE≥22 and had the ability to understand and perform therapeutic commands. All participants read and signed consent form before starting the project and ethical approved was obtained from the OT Research committee (IUMS). Before starting therapeutic sessions, level of performance of activities of daily living (Barthel Index), (Modified Rankin Scale) and participation (Canadian Occupational Performance Measure) of participants were measured. Interview and evaluation process was experienced therapist.

### 2.2. Assessment tools

The three tools used in this study are described subsequently. Barthel Index (BI) is very commonly used to evaluate the level of performance of activities of daily living. This index has ten items which includes the main activities of daily living. It has a good reliability and validity in patients with cerebrovascular accident. Ten items Include self-care activities (eating, bathing, personal hygiene, bowel and bladder care, dressing and toileting) and mobility (transfer, ambulation and stair climbing). The maximum score is 100 which represent the full independence. This index is valid and reliable tool in Persian language (Sulter et al., 1999; Oveisgharan et al., 2006).

The Modified Rankin Scale (MRS) is used for assessing global disability and stroke complications and is predicted functional recovery after stroke. It is graded from 0 to 5 where 0 represents no disease symptoms and 5 represents severe impairment (Banks et al., 2007; Lai et al., 2001; Ghandehari et al., 2012).

The Canadian Occupational Performance Measure (COPM) is used to determine changes based on patient understanding, before and after the interventions used in occupational therapy. This tool was designed on the basis of occupational therapy guidelines in client-centered activity. The COPM is described based on individual problems in three areas; leisure, productivity and self-care and is completed form semi-structured interview. These problems are those one can do it or need to do it or had the ability to do it before, but now can’t perform it or do not satisfy with it. The importance of each activity is scored from 1 to 10, so that below the 10, main priority is placed. By priorities classification, individuals score their performance and their satisfaction of the performance from 1 to 10. This measure is completed before and after the intervention sessions and changes in the performance and satisfaction scores are reported as a result of the intervention (Atashi et al., 2010; Chan et al., 1997; Dedding et al., 2004; Cup et al., 2003).

### 2.3. Procedure

The CCT approach was designed for people with chronic stroke with the aim of improving the level of performance of daily activities and also adapting implements and facilitating performance in series of tasks. These group tasks were scheduled for 6 sessions of 120 minutes with the emphasis on the use of affected side in doing activities, use of adaptive equipment and association with other members of the group. Main activities were divided into three areas; mobility exercises of the upper and lower extremities (20 minutes in accordance with standard practice, such as increasing the repetition, duration and complexity of the task), craft activities (40 Minutes include making a photo frame, coloring, ceramics, sculpturing) and activities related to cooking and eating (40 minutes including cutting, peeling off, cooking, move dining utensils and eating Independently).

Group sessions held in the activities of daily living clinic in occupational therapy (OT) department. This clinic is a simulated residential unit with the same layout and type of equipment. Movement exercises and craft activities were performed in the lounge of one of the rooms and activities related to cooking were done in the kitchen of the clinic. Each session began with the introduction of the group members and explanation of the session goals by occupational therapists and during the session therapists guided the group to achieve the therapeutic targets. For selecting craft and cooking activities all participants were shared their experiences and therapist provided adaptation and facilitation before the start of each session. The leaders included two occupational therapists (MSc. OT) and one supervisor (PhD OT). The control group received traditional OT interventions individually by an occupational therapist in outpatient clinic.

### 2.4. Analysis

The Shapiro-Wilk test was used to assess the normality of data distribution with P value>0.05. All scores were normally distributed; expect BI score. Therefore a logarithmic transformation was used for BI score (Harwell et al., 1992).

To evaluate outcomes, the differences in COPM (Performance/Satisfaction), MRS, BI scores between the treatment and control groups in pre-post intervention were analyzed. Paired-t test was used to calculate before and after the intervention results in two groups. The effects of time (pre and post treatment) and group (treatment and control) on the measurements (main effects and interaction of these effects) were analyzed using a 2×2 (group×time) mixed model analysis of variance (ANOVA). The Bonferroni adjustment method was used for multiple comparisons. Statistical significance was set at P<0.05.

## 3. Results

There were 7 participants in treatment and control, most of them were right hemiplegia, and majorities were married. Demographic characteristics of the participants are reported in Table 1. Table 2 represents a summary of ANOVA results for all the measurements. The main effects of time were significant for all tests. The main effects of group were not significant for all tests. Interactions of time by group were significant for COPM (performance/satisfaction). Before and after the intervention scores of MRS, BI, COPM (Performance/Satisfaction) are shown in Table 3.

**Table 1:** General characteristics of subjects.

**Characteristics**		**Treatment (N=7)**	**Control (N=7)**
Mean age±SD (years)		49 ±9.66	55±10.1
Mean time since stroke±SD (months)		19±15.04	18±14.02
MMSE (mean)		27	25
Male gender		N=3(43%)	N=6(85%)
Affected side	Right	N=6(86%)	N=6(86%)
	Left	N=1(14%)	N=1(14%)
Education	Secondary school	N=3(43%)	N=5(71%)
	University	N=4(57%)	N=2(29%)
Vocational status	Home maker	N=2(29%)	N=1(14%)
	Employed	N=2(29%)	N=1(14%)
	Retired	N=3(43%)	N=5(71%)
Marital status	Married	N=6(86%)	N=7(100%)

**Table 2:** Summary of analysis of variance for measurements (N=14).

	**Values**		**df**	**F**	**Sig.**	**Effect Size**
Barthel index	Main effect	Time	1	42.03	0.000**	0.750
		Group	1	0.07	0.521	0.040
	Interaction effect	Time*Group	1	0.70	0.861	0.005
MRS	Main effect	Time	1	6.18	0.023**	0.362
		Group	1	0.62	0.446	0.049
	Interaction effect	Time*Group	1	0.27	0.611	0.022
Performance	Main effect	Time	1	39.16	0.000**	0.765
		Group	1	0.03	0.867	0.002
	Interaction effect	Time*Group	1	14.56	0.002**	0.548
Satisfaction	Main effect	Time	1	17.60	0.001**	0.595
		Group	1	0.01	0.973	0.000
	Interaction effect	Time*Group	1	14.22	0.003**	0.542

**Table 3:** Independent t-test for pre and post treatment between groups (N=14).

**Assessment**	**Groups**	**F**	**Sig.**
Barthel index	Pre (treatment-control)	1.60	0.312
	Post (treatment-control)	0.82	0.380
MRS	Pre (treatment-control)	4.32	0.061
	Post (treatment-control)	1.96	0.187
Performance	Pre (treatment-control)	2.27	0.158
	Post (treatment-control)	2.62	0.131
Satisfaction	Pre (treatment-control)	3.33	0.931
	Post (treatment-control)	9.49	0.010

According to Table 2, COPM scale changes in the “Performance” and “Satisfaction” scores in treatment group and “Performance” score in control group were significant (P<0.05), but “satisfaction” scores in control group was not significant (P>0.05). The MRS scale in two groups; treatment and control; significant level with P>0.05, it means, there were no change in the level of the disease. Improvement in the performance of activities of daily living assessed by BI, show significant difference in two groups (P<0.05).

## 4. Discussion

This study was performed to evaluate changes in the performance of activities of daily living, level of the disability and satisfaction of the performance after the group therapy intervention with emphasis on everyday activities in people with stroke. The results indicated improvement in the performance of activity of daily living and the satisfaction level of performance. In this regard, Jorgensen (1995) with a study of stroke patients in the acute phase, showed that in 95% of patients neurological improvement achieved in the first 11 weeks after stroke and the best performance of activities of daily living is achieved in 12.5 weeks after stroke.

After this period, people with chronic stroke nearly accomplish a constant level of improvement in the activities of daily living (Guidetti et al., 2002). Rehabilitation interventions can have an impact on improving ADL. Occupational therapy is considered as an essential part in rehabilitation after stroke. The main goal of occupational therapy is the use of activity or targeted interventions to achieve the maximum level of independence. Increase the level of independence in activities of daily living, is an important criterion to evaluate the effectiveness of rehabilitation after stroke, which is commonly used as a result in the trials (Legg et al., 2007).

Jinuk (2013) revealed that task-oriented activities can improve the level of activities of daily living (by using modified Barthel Index) (Choi et al., 2013). Also Ko (2015), confirmed the impact of these training on motor recovery and ADL scores by the Assessment of Motor and Process Skills scale (AMPS) (KO et al., 2015). Steultjens and his colleagues study (2003) showed no effect of sensorimotor exercises and CCT on improving ADL and IADL. The score of BI in two groups were significant but in treatment group the average difference was higher than control group. According to the purpose of groups in this study which is based on emphasis on performing activities of daily living (such as eating, mobility, crafts and cooking) and the use of adaptive devices and bimanual techniques to do tasks, there may be some patients who do not have an experience of using this type of functional exercises over the time. It can be expected that according to the results the participants demonstrate improvement in the level of activities of daily living at the end of the sessions.

The Modified Rankin Scale is often used as a final assessment in RCT studies in patients with stroke to determine the level of disability caused by stroke. According to the study with the aim of MRS usage in RCT studies by Banks (2006) and his colleagues, it was shown that although in people with stroke MRS score changes may not be achieved significant in grade≤2; these changes may clinically be significant. Stroke Impact Scale was used in KO (2015) study to assess the changes in the level of disability and quality of life, so that there was no significant change in level of disability aligns with the results of this study. Despite average scores in two groups, the MRS scores had no significant changes. That it showed clinically significant changes but not statistically.

In client-centered approach, people play an active role in the process of treatment based on prioritization of their problems in the areas of self-care, productivity and leisure. In patients with stroke, the resumption of important activities which they were independent in doing it before stroke can be improved by changes in type of the activity and use of adaptive equipment (Egan et al., 2007; Morgan et al., 2002). The main goal of most CCT interventions is to improve the level of performance and mobility in people with stroke. In “Performance” scores in COPM in two groups’ significant changes were obtained but in treatment group average differences had been more than control group. Improvement in “Performance” was remarkable due to the emphasis on activity/occupation-based tasks, use of adaptive equipment and modification in implementation of activities in group. The change in “Satisfaction” scores of treatment group was significant and notable; despite in control group wasn’t significant.

Moreover, the level of people’s expectations for implementation of activities before stroke was altered, that can be observed by increased changes in the “Satisfaction” scores of performance. Findings of this part of the intervention are similar to that of Morgan and Bodaim. Quality of life is the ways a person can realize about objectives, expectations, interests, values individually in his life. Quality of life for patients is associated with their level of life satisfaction. Since in this study the level of satisfaction of Implementation had improved, it assumed that quality of life related to everyday activity was improved too. Non-linear relations between the components of the organization in the dynamic system affect outcomes, and led to self-organization. This phenomenon refers to the system’s ability to control and organize its performance, so that the system organize his performance spontaneously in order to be responsive to the requirements imposed on the system in the best possible way. This feature allows the system to compromise and adapt to the changes. This new view is also called bio dynamic or non-linear dynamic biology, clarifies the ambiguity in various fields of cellular molecular biology to neuroscience.

Two-way relationships were formed between participation in occupation and neuro-dynamic system in the brain can impact the quality of brain self-organization. (Lazzarini et al., 2004; Derakhshanrad et al., 2015). It seems that performing desired activity can affect quality of life and life satisfaction. Doing favorite craft activities and inclusion of purposeful repetitive movements of the upper limb with the necessary adaptations in order to being successful in the activity can increase awareness and insight, communication and Interpersonal interactions and provide appropriate emotional responses. Limitations of this study include absence of direct measurement tool for assessing changes in craft activities and also lack of measurement of mood changes in participants.

The present study showed that activities of daily living, crafts, and mobility in the treatment group can affect the performance and satisfaction of stroke survivors. In people with stroke, meaningfulness, i.e. choice of activities, can affect self-organization and reconstruction of the brain. However, this study requires further research.
